# Supplementary material for: Structural basis for the SUMO protease activity of the atypical ubiquitin-specific protease USPL1
Source: Nat Commun. 2022 Apr 5;13:1819. doi: 10.1038/s41467-022-29485-0 (PMC8983731; doi:10.1038/s41467-022-29485-0)
Supplement: Supplementary file 1 — Supplementary Information [file 41467_2022_29485_MOESM1_ESM.pdf]

Supplementary information

Structural basis for the SUMO protease activity of the atypical ubiquitin-specific protease USPL1

Ying Li[1], Nathalia Varejão[1] and David Reverter[1*]

[1]Institut de Biotecnologia i de Biomedicina (IBB) and Dept. de Bioquímica i Biologia Molecular, Universitat Autònoma de Barcelona, 08193 Bellaterra, Spain.
*Correspondence: david.reverter@uab.cat

Supplementary information Inventory:
Supplementary Table 1
Supplementary Figures 1-9
Supplementary References

Supplementary Table 1 – List of the used primers.

USPL1CD fw	CGCGGATCCATGCCACTGGAGAGGAAATG
USPL1CD rv	AAAGCGGCCGCTTAAAGTGGAAGGCAGGC
Δ14SUMO2GCVY fw	CGGCCTGGTGCCGCGCGGCAGCCATAACGATCATATTAATTTGAAGGTG
Δ14SUMO2GCVY rv	GACGGAGCTCGAATTCGGATCCCTAGTAGACACATCCCGTCTGCTGTTGG
USPL1 Phe335 to Ala fw	AGGTGATATGGAAAGCCCTGTGGCTGCATTTCCCCTGCTCTT
USPL1 Phe335 to Ala rv	AAGAGCAGGGGAAATGCAGCCACAGGGCTTTCCATATCACCT
USPL1 Arg324 to Ala fw	TTAGCCTTCAGCCCCAGCTTGCATGCACATTAGGTGATAT
USPL1 Arg324 to Ala rv	ATATCACCTAATGTGCATGCAAGCTGGGGCTGAAGGCTAA
USPL1 Met330 to Ala fw	AGATGCACATTAGGTGATGCGGAAAGCCCTGTGTTTGCAT
USPL1 Met330 to Ala rv	ATGCAAACACAGGGCTTTCCGCATCACCTAATGTGCATCT
USPL1 Asn398 to Ala fw	CCATTTTGGTCCATGTGCCAATTGCAACAGTAAATCACAAAT
USPL1 Asn398 to Ala rv	ATTTGTGATTTACTGTTGCAATTGGCACATGGACCAAAATGG
USPL1 Tyr451 to Phe fw	AACTTCTGTAATTCAGTTTCGAGCAAATAATCATTTTATAACAT
USPL1 Tyr451 to Phe rv	ATGTTATAAAATGATTATTTGCTCGAAACTGAATTACAGAAGTT
USPL1 His493 to Asn fw	AGTTCCTGCTTCAGAGATAAATATTGTTATTTGGGAAAG
USPL1 His493 to Asn rv	CTTTCCCAAATAACAATATTTATCTCTGAAGCAGGAACT
USPL1 His421 to Asn fw	ATCTCCCATATTCATGTTGAACTTTGTAGAAGGCTTACC
USPL1 His421 to Asn rv	GGTAAGCCTTCTACAAAGTTCAACATGAATATGGGAGAT
USPL1 Glu331 to Pro fw	TGCACATTAGGTGATATGCCAAGCCCTGTGTTTGCATTT
USPL1 Glu331 to Pro rv	AAATGCAAACACAGGGCTTGGCATATCACCTAATGTGCA
USPL1 Phe457 to Tyr fw	CAGTATCGAGCAAATAATCATTATATAACATGGATTTTAGATGCT
USPL1 Phe457 to Tyr rv	AGCATCTAAAATCCATGTTATATAATGATTATTTGCTCGATACTG
USPL1 Phe457 to Leu fw	CAGTATCGAGCAAATAATCATCTTATAACATGGATTTTAGATGCT
USPL1 Phe457 to Leu rv	AGCATCTAAAATCCATGTTATAAGATGATTATTTGCTCGATACTG

a

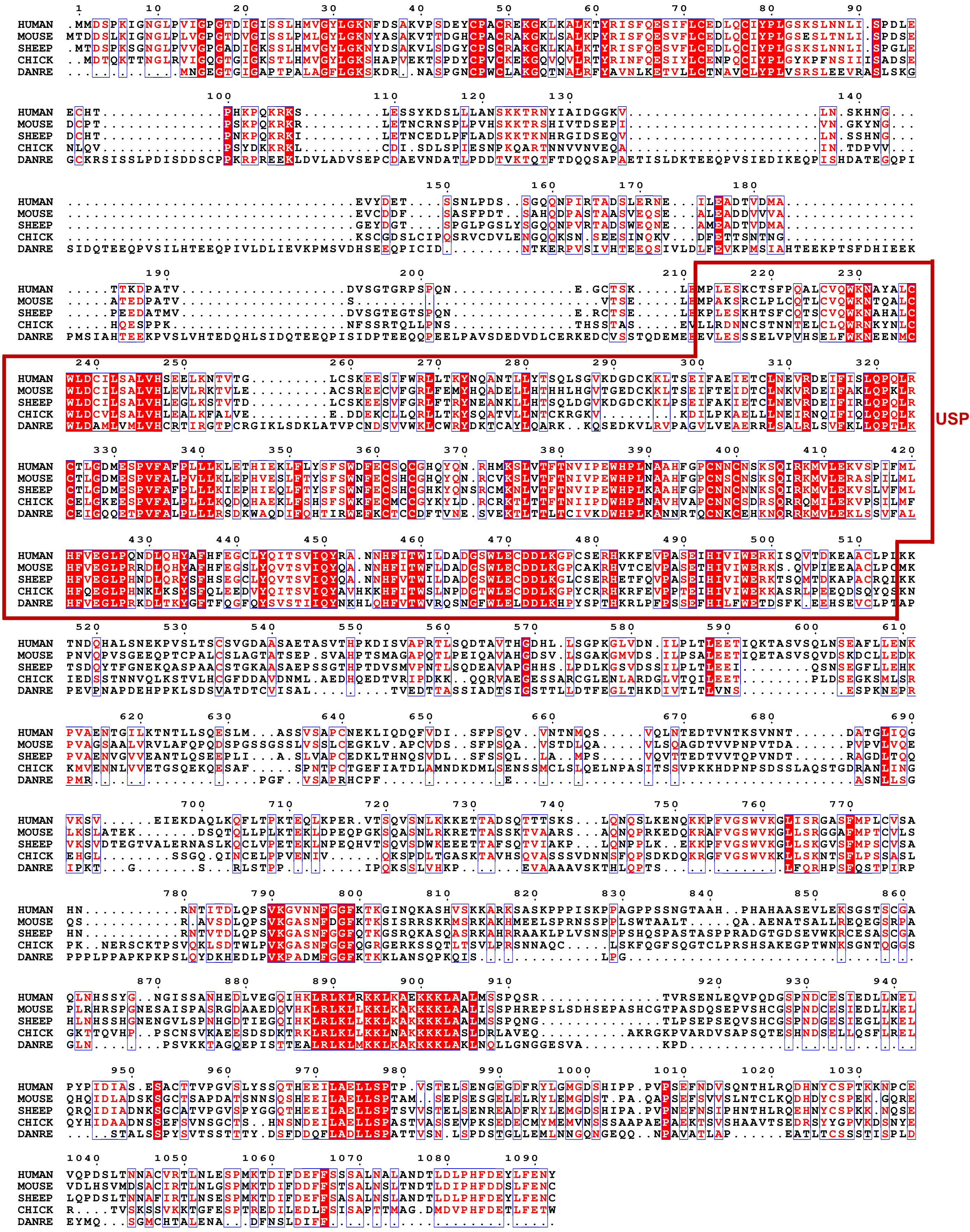

b

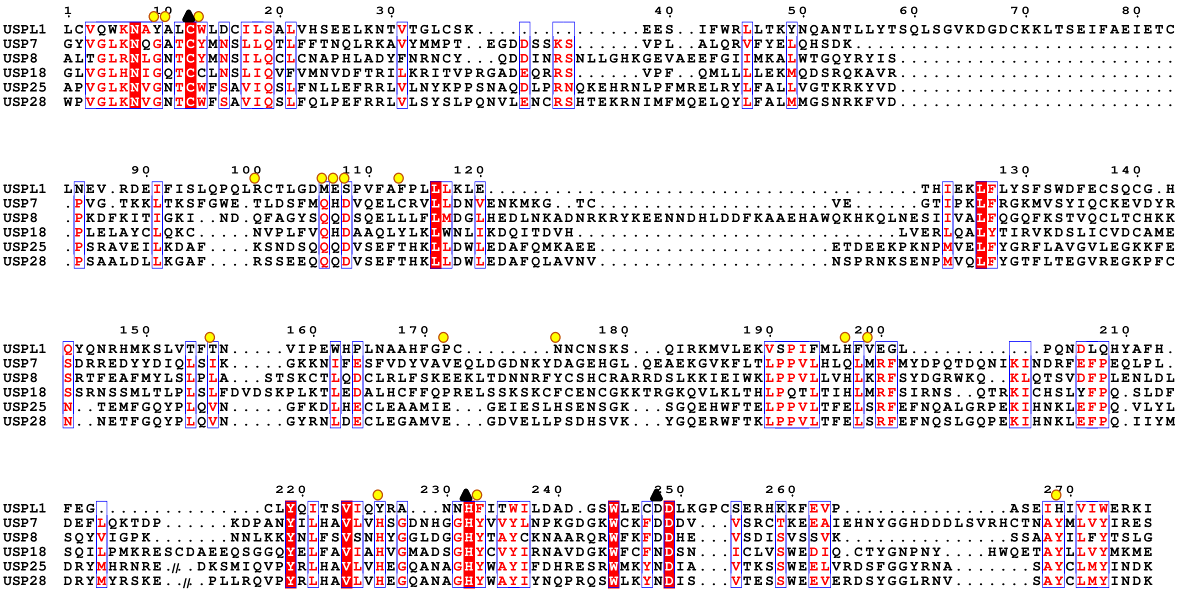

c

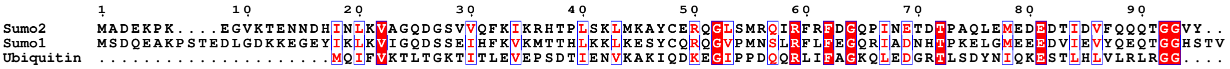

Supplementary Figure 1

(a) Multiple sequence alignment of USPL1 with its orthologs in mouse, sheep, chicken and danio rerio (zebrafish). Red represents high conservation. USP-like catalytic domain is labeled by a red frame. (b) Structural/sequential alignment of the USPL1 catalytic domain with the catalytic domains of USP7, USP8 USP28, USP18, USP25 and USP28. Blue triangles represents the catalytic triad and yellow circles the contact residues with SUMO2 (c) Sequence alignment of SUMO1, SUMO2 and ubiquitin. All sequence alignments are from Clustal Omega and formatted by ESPript [1].

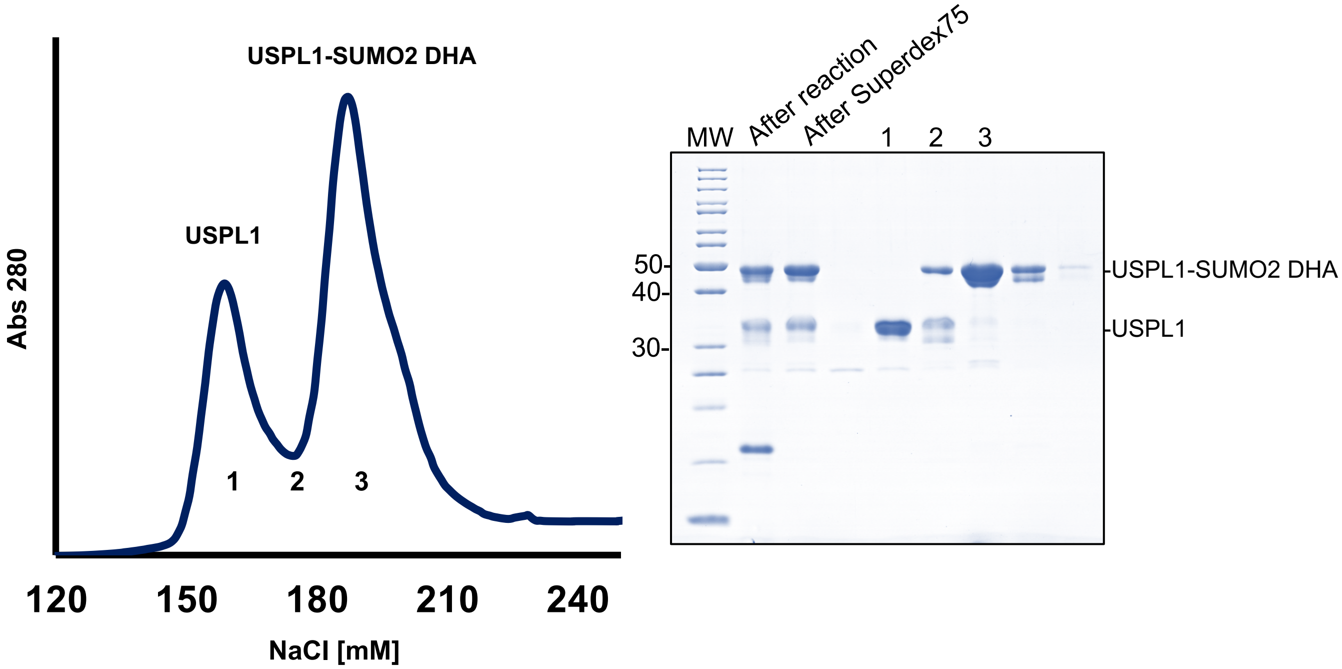

Supplementary Figure 2. Purification of the USPL1-SUMO2 complex

Left, anionic exchange chromatography profile of the purification of the USPL1-SUMO2 complex after the incubation of the SUMO2 DHA precursor with USPL1 catalytic domain at 37 °C for 2 hours. *Right*, uncropped SDS-PAGE of the indicated fractions of the anion exchange column. n=1 technical replicate.

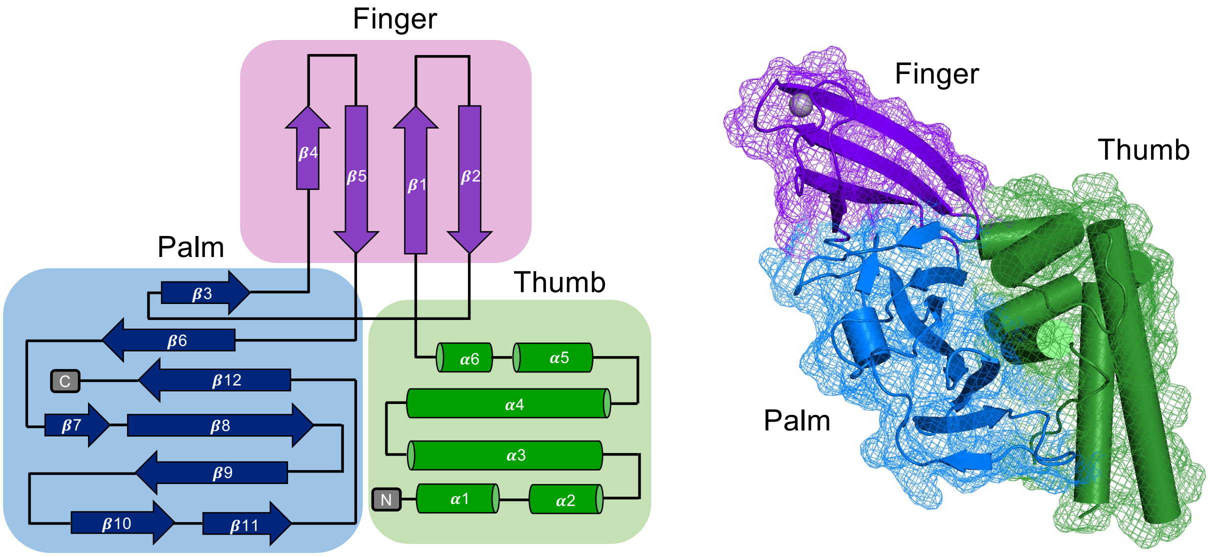

Supplementary Figure 3. Topology diagram of USPL1.

The topology diagram of the USPL1 right hand-like subdomains: *Finger* (purple), *Palm* blue), and *Thumb* (green). The cartoon representation of USPL1 is presented in the same colors.

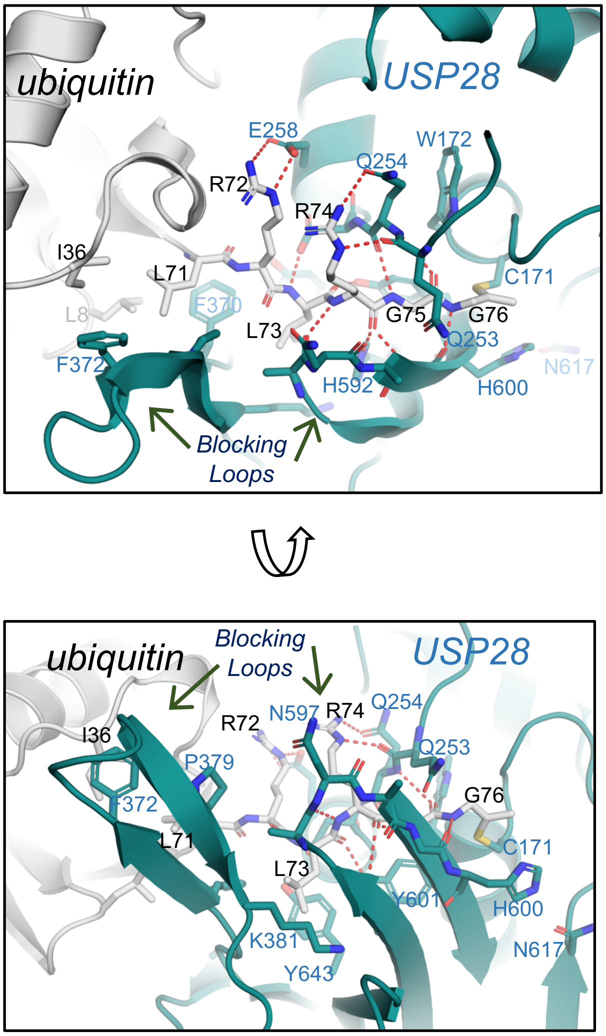

Supplementary Figure 4. USP28-ubiquitin interface details.

Two views of the stick representation of the main contacts of the C-terminal of ubiquitin in complex with the active site groove of USP28 (PDB code 6HEK)[2]. USP28 catalytic domain and ubiquitin are shown in green and grey, respectively. Ubiquitin and USP28 interface residues are labelled. Dashed lines indicate hydrogen bond contacts.

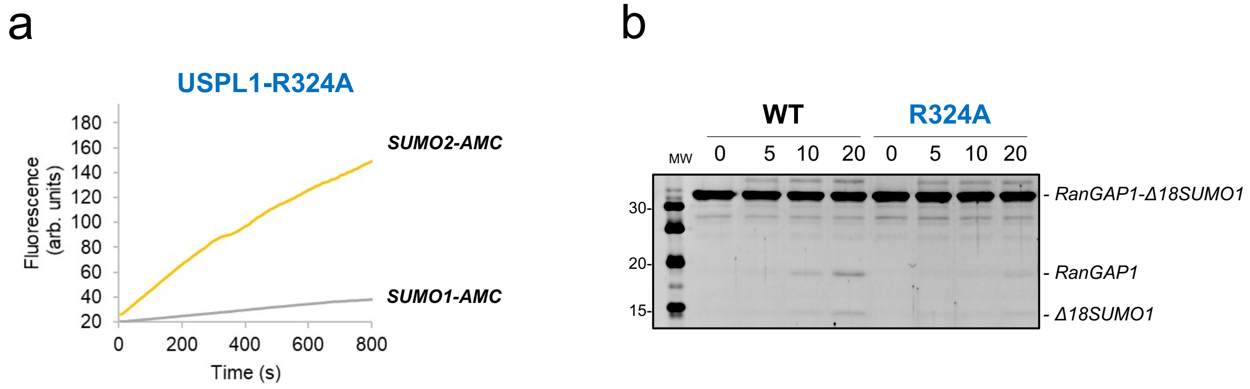

Supplementary Figure 5. Catalytic activity of the USPL1 R324A mutant. **(a)** Activity assay of USPL1 R324A mutant of the C-terminal tail interface using the SUMO2-AMC and SUMO1-AMC substrates. SUMO-AMC (0.25 μM) was incubated with 1nM of USPL1 mutant at 30 °C, and released AMC was detected by fluorescence. **(b)** Time course assays of 20 nM USPL1 wild type and R324A mutant using 1 μM RanGAP1-Δ18SUMO1 substrate at 37 °C. n=1 technical replicate. Uncropped gel is shown in the Source Data file.

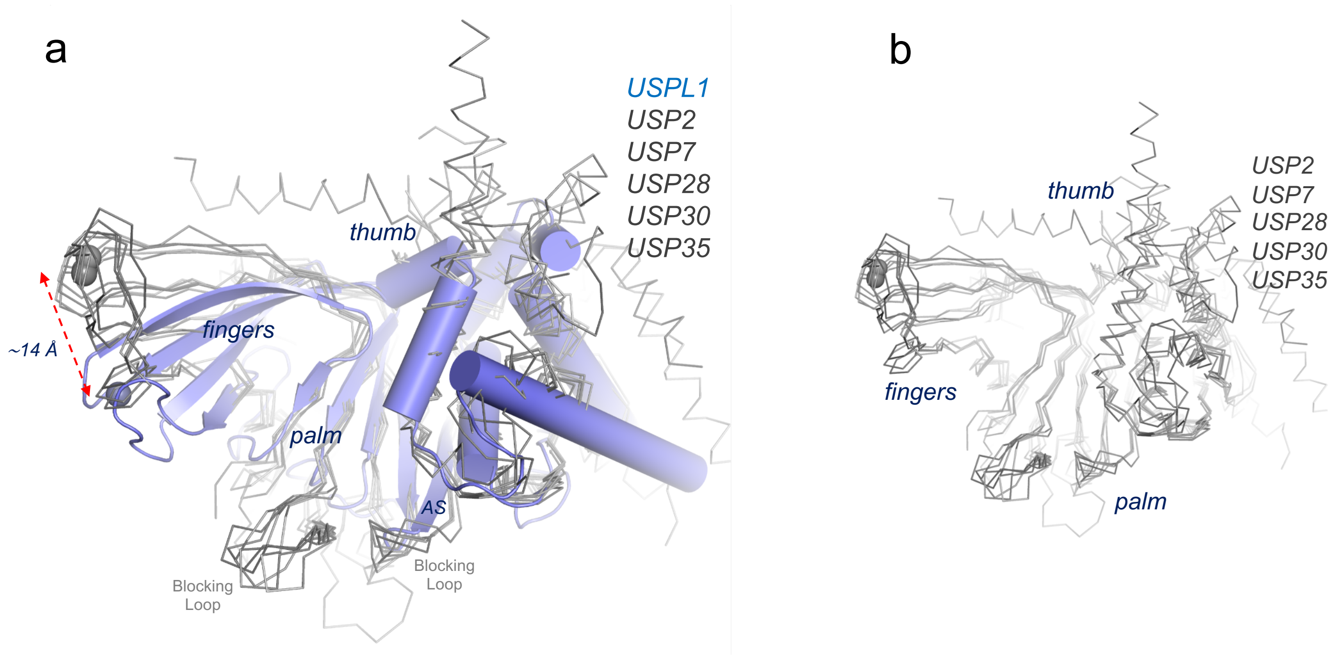

Supplementary Figure 6. Structural overlapping of USPL1 with ubiquitin USPs.

(a) Multiple structural overlapping of USPL1 with five structures of USP in complex with ubiquitin. USPL1 is shown in a blue cartoon representation and the five ubiquitin USPs in grey ribbon representation. Double red arrow indicates the average distance between the Zn^{2+} atom in USPL1 compared to the Zn^{2+} in ubiquitin USPs. USP subdomains are labelled. (b) Multiple structural overlapping of five structures of USP in complex with ubiquitin. Ubiquitin has been removed from the picture. The USP structures correspond to USP2 (PDB code 2hd5), USP7 (PDB code 5jtv), USP28 (PDB code 6hek), USP30 (PDB code 5ohk), USP35 (PDB code 5txk), USP45 (PDB code 5l8h) and USPL1 (PDB code 7p99) [2-7].

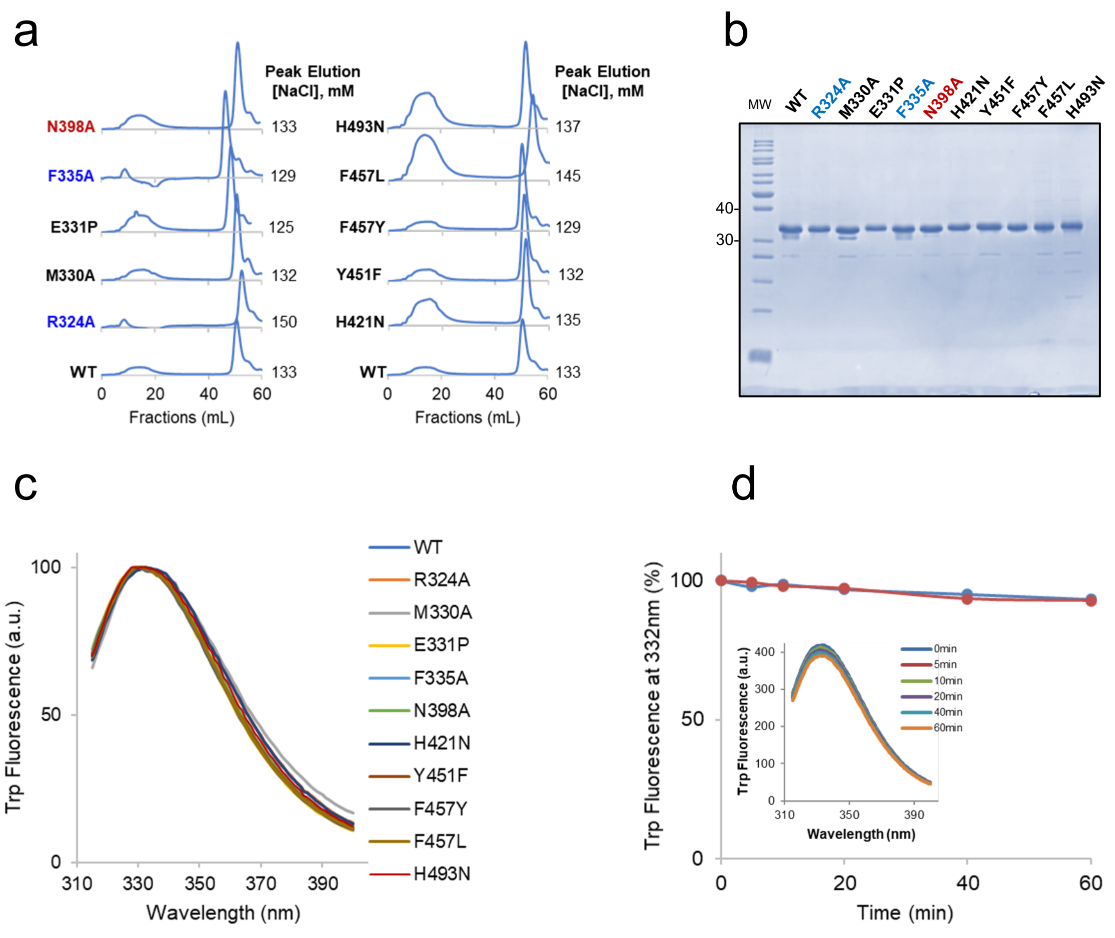

Supplementary Figure 7. Purification and Trp-fluorescence of USPL1 mutants. (a) Resource Q elution profile of the purification of USPL1; NaCl concentration where the major peak eluted is shown. (b) SDS-PAGE showing the collected RQ peaks. n=1 technical replicate. Uncropped gel shown in the Source Data file. (c) Intrinsic fluorescence showing that all mutants are well-folded. (d) Stability of WT (blue) and H493N mutant (red) at 30°C; the inset shows the raw Trp spectra used to calculate stability. Source data provided as a Source Data file.

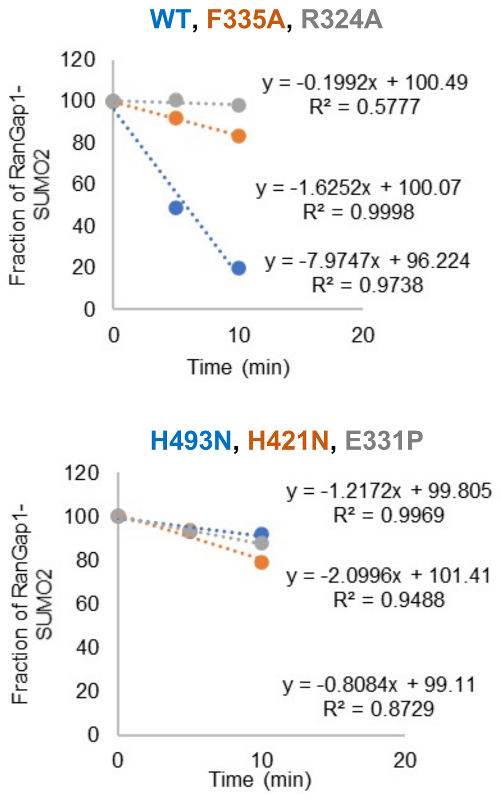

Supplementary Figure 8. **Raw data *for RanGAP1-SUMO2 deconjugation.*** Source data provided as a Source Data file.

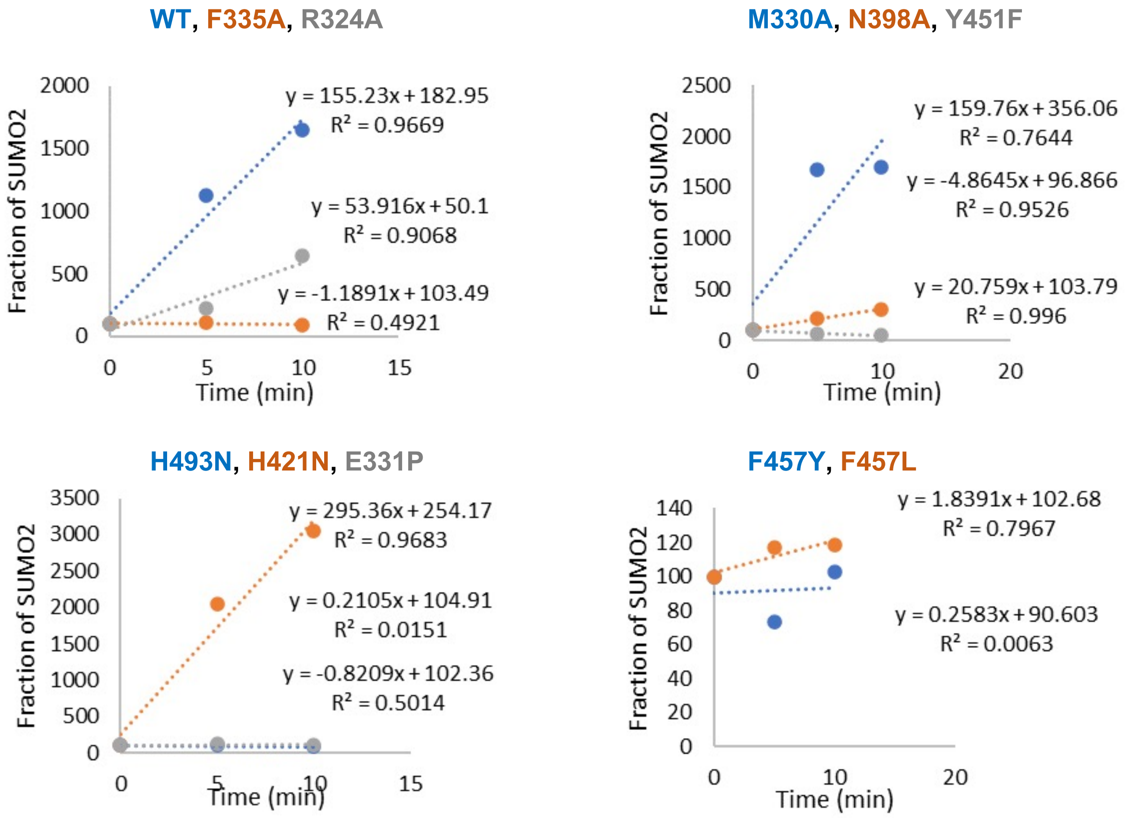

Supplementary Figure 9. **Raw data *for di-SUMO2 deconjugation.*** Source data provided as a Source Data file.

Supplementary References

1. Robert, X. & Gouet, P. Deciphering key features in protein structures with the new ENDscript server. *Nucleic Acids Res.* **42**, W320-4 (2014).

2. Gersch, M. *et al.* Distinct USP25 and USP28 Oligomerization States Regulate Deubiquitinating Activity. *Mol. Cell* **74**, 436-451.e7 (2019).

3. Renatus, M. *et al.* Structural basis of ubiquitin recognition by the deubiquitinating protease USP2. *Structure* **14**, 1293–1302 (2006).

4. Rougé, L. *et al.* Molecular Understanding of USP7 Substrate Recognition and C-Terminal Activation. *Structure* **24**, 1335–1345 (2016).

5. Gersch, M. *et al.* Mechanism and regulation of the Lys6-selective deubiquitinase USP30. *Nat. Struct. Mol. Biol.* **24**, 920–930 (2017).

6. Leznicki, P. *et al.* Expansion of DUB functionality generated by alternative isoforms - USP35, a case study. *J. Cell Sci.* **131**, (2018).

7. Dharadhar, S., Clerici, M., van Dijk, W. J., Fish, A. & Sixma, T. K. A conserved two-step binding for the UAF1 regulator to the USP12 deubiquitinating enzyme. *J. Struct. Biol.* **196**, 437–447 (2016).
